# Editorial: FGF21 as a therapeutic target for obesity and insulin resistance: from rodent models to humans

**DOI:** 10.3389/fendo.2023.1253675

**Published:** 2023-08-07

**Authors:** A. L. De Sousa-Coelho, R. Rodriguez-Rodriguez, S. Softic, J. W. Jonker, J. Relat

**Affiliations:** ^1^ Escola Superior de Saúde (ESS), Universidade do Algarve (UAlg), Faro, Portugal; ^2^ Algarve Biomedical Center Research Institute (ABC-RI), Universidade do Algarve (UAlg), Faro, Portugal; ^3^ Algarve Biomedical Center (ABC), Faro, Portugal; ^4^ Basic Sciences Department, Faculty of Medicine and Health Sciences, Universitat Internacional de Catalunya, Barcelona, Spain; ^5^ Centro de Investigación Biomédica en Red de Fisiopatología de la Obesidad y la Nutrición (CIBEROBN), Instituto de Salud Carlos III, Madrid, Spain; ^6^ Department of Pediatrics, Division of Pediatric Gastroenterology, Kentucky Children’s Hospital, and Department of Pharmacology and Nutritional Sciences, University of Kentucky College of Medicine, Lexington, KY, United States; ^7^ Department of Pediatrics, University of Groningen, University Medical Center Groningen, Groningen, Netherlands; ^8^ Department of Nutrition, Food Sciences and Gastronomy, School of Pharmacy and Food Sciences, University of Barcelona, Santa Coloma de Gramenet, Spain; ^9^ Institute of Nutrition and Food Safety of the University of Barcelona (INSA-UB), Santa Coloma de Gramenet, Spain

**Keywords:** FGF21 (fibroblast growth factor 21), obesity, insulin resistance, metabolism, beta-Klotho

Obesity is a global pandemic that requires the urgent development of therapies and prevention strategies. To define new pharmacologic therapies or nutritional approaches it is mandatory to find new targets. Fibroblast growth factor 21 (FGF21) is considered a potential target to treat obesity, due to its favorable metabolic activity, signalling pathways and regulatory mechanisms. It is well-documented that FGF21 is induced by a wide range of biological stress conditions and a key signal that communicates and coordinates the physiologic response to restore the metabolic homeostasis in different tissues (1). FGF21 is elevated in pathological conditions such as obesity, insulin resistance, or fatty liver disease where an impairment of its signalling has been described (2). On the other hand, FGF21 analogues tested in overweight/obese patients with type 2 diabetes or NAFLD/NASH can reduce dyslipidaemia and steatosis, but improvements in glycaemic control or body weight were not globally restored (3). This suggests that pharmacologic effects of FGF21 are different from its physiological effects. In this Research Topic “*FGF21 as a therapeutic target for obesity and insulin resistance: from rodent models to humans*”, we include publications related to new advances involving FGF21, its signalling pathway, and its potential as a target to treat obesity.

Manuscript by Spann et al. highlighted the function of endogenous FGF21 in metabolism. In this thorough review the authors discuss many stimuli involved and which tissue is either the source of or the target of FGF21, bringing into discussion relevant data from over a decade of research (Spann et al.).

Previous studies in humans showed the involvement of FGF21 in dietary preference, appetite, and plasma lipids (4–6). Genetic studies in humans have associated some single nucleotide polymorphisms (SNPs) in and around the FGF21 gene with carbohydrates, protein, fat, and alcohol preference (7–9). Qian et al. systematically reviewed the association between lifestyle and circulating FGF21 levels. Including a total of 50 studies, this meta-analysis identified many disperse stimuli that significantly upregulated FGF21 levels such as smoking, alcohol consumption, acute exercise, hypercaloric carbohydrate- or fat-rich diet, amino acid or protein restriction, or excessive fructose intake (Qian et al.). Serum FGF21 is proposed to be a biomarker to assess the effects of different lifestyle interventions and metabolic interactions.

Beta-Klotho (KLB), a co-receptor for FGF21, is an important regulator of FGF21 action (10–13). Beyond the liver and adipose tissue, in humans, KLB is also detected in the breast, bone marrow, and pancreas and these differences may explain, at least in part, the differential metabolic effects of FGF21 in humans (14). In this Research Topic, Aaldijk et al. reviewed both biological and pharmacological tissue-dependent functions of KLB. This review comprehensively describes the importance of the identified genetic KLB variants and their phenotypic associations. Furthermore, it discusses the regulation of both transcript and protein KLB expression levels in different metabolic tissues and its response to different stimuli. Finally, the most recent data regarding KLB-targeting drugs in clinical studies is depicted, including FGF19 and FGF21 analogues, and specific KLB/FGFR1c agonists (Aaldijk et al.).

Many questions are still open regarding the metabolic role of FGF21 in humans, especially under nonpathological conditions. Crudele et al. calculated a cut-off for total serum levels of FGF21 according to visceral adiposity, which identified subjects with fasting hyperglycaemia. Intriguingly, waist circumference correlated with total FGF21 serum levels but did not correlate with intact functional FGF21. This suggests that an increase in total FGF21 but not functional FGF21 predicts dysmetabolic conditions, highlighting the complexity of its mechanism of action.

It is worth noting that some divergent data between mice and humans have been reported regarding the mechanisms that induce FGF21 expression and on its metabolic effects. While in mice short-term fasting and ketogenic diets increase FGF21 serum levels, in humans this induction was observed only after a very prolonged period of fasting (7–10 days) (1). Nevertheless, studies using animal and cellular models to explore FGF21 signalling in central and peripheral tissues are crucial for understanding the upstream and downstream molecular mechanisms of the metabolic effects of FGF21, including its receptors and regulating proteins. In a study using the obesity-resistant uncoupling protein 1 (UCP1) knock-out (KO) mice, Hazebroek and Keipert elegantly reported improved sensitivity to exogenous FGF21 in the UCP1 KO mice after long-term high fat diet feeding. Remarkably, the increased FGF21 sensitivity was observed in inguinal white adipose tissue (iWAT) but not in liver, suggesting iWAT as the key tissue regulating FGF21-mediated metabolic improvements.

The articles included in this Research Topic provide evidence of new advances involving FGF21, including molecular data from animal models but also from humans, as well as reviews that comprehensively highlight the new findings, shedding light on the real potential of FGF21 as a target in the treatment of obesity and related metabolic diseases.

## Author contributions

ALDS-C: Investigation, Writing – original draft, Writing – review & editing. RR-R: Conceptualization, Investigation, Writing – review & editing. SS: Investigation, Writing – review & editing. **JJ:** Investigation, Writing – review & editing. JR: Conceptualization, Investigation, Project administration, Writing – original draft, Writing – review & editing.
